# The kringle IV type 2 domain variant 4925G>A causes the elusive association signal of the *LPA* pentanucleotide repeat

**DOI:** 10.1016/j.jlr.2022.100306

**Published:** 2022-10-26

**Authors:** Rebecca Grüneis, Hansi Weissensteiner, Claudia Lamina, Sebastian Schönherr, Lukas Forer, Silvia Di Maio, Gertraud Streiter, Annette Peters, Christian Gieger, Florian Kronenberg, Stefan Coassin

**Affiliations:** 1 Department of Genetics, Institute of Genetic Epidemiology, Medical University of Innsbruck, Innsbruck, Austria; 2 German Center for Diabetes Research (DZD), München-Neuherberg, Germany; 3 Institute of Epidemiology, Helmholtz Zentrum München - German Research Center for Environmental Health, Neuherberg, Germany; 4 Department of Epidemiology, Institute for Medical Information Processing, Biometry and Epidemiology, Ludwig-Maximilians-University Munich, Munich, Germany; 5 Research Unit of Molecular Epidemiology, Helmholtz Zentrum München, German Research Center for Environmental Health, Neuherberg, Germany

**Keywords:** lipoprotein(a), lipoprotein(a) metabolism, apolipoproteins, genomics, lipoproteins, apolipoprotein(a), short tandem repeat, lipoprotein(a) regulation, complex genome region, Apo(a), apolipoprotein(a), HMW, high-molecular-weight, LD, linkage disequilibrium, LMW, low-molecular-weight, Lp(a), lipoprotein(a), KIV, kringle IV, KORA, Cooperative Health Research in the Region of Augsburg, PNR, pentanucleotide repeat, STR, short tandem repeat

## Abstract

Lipoprotein(a) [Lp(a)] concentrations are regulated by the *LPA* gene mainly via the large kringle IV-type 2 (KIV-2) copy number variation and multiple causal variants. Early studies suggested an effect of long pentanucleotide repeat (PNR) alleles (10 and 11 repeats, PNR10 and PNR11) in the *LPA* promoter on gene transcription and found an association with lower Lp(a). Subsequent in vitro studies showed no effects on mRNA transcription, but the association with strongly decreased Lp(a) remained consistent. We investigated the isolated and combined effect of PNR10, PNR11, and the frequent splice site variant KIV-2 4925G>A on Lp(a) concentrations in the Cooperative Health Research in the Region of Augsburg F4 study by multiple quantile regression in single-SNP and joint models. Data on Lp(a), apolipoprotein(a) Western blot isoforms, and variant genotypes were available for 2,858 individuals. We found a considerable linkage disequilibrium between KIV-2 4925G>A and the alleles PNR10 and PNR11. In single-variant analysis adjusted for age, sex, and the shorter apo(a) isoform, we determined that both PNR alleles were associated with a highly significant Lp(a) decrease (PNR10: β = −14.43 mg/dl, 95% CI: −15.84, −13.02, *P* = 3.33e-84; PNR11: β = −17.21 mg/dl, 95% CI: −20.19, −14.23, *P* = 4.01e-29). However, a joint model, adjusting the PNR alleles additionally for 4925G>A, abolished the effect on Lp(a) (PNR10: β = +0.44 mg/dl, 95% CI: −1.73, 2.60, *P* = 0.69; PNR11: β = −1.52 mg/dl, 95% CI: −6.05, 3.00, *P* = 0.51). Collectively, we conclude that the previously reported Lp(a) decrease observed in pentanucleotide alleles PNR10 or PNR11 carriers results from a linkage disequilibrium with the frequent splicing mutation KIV-2 4925G>A.

Lipoprotein(a) [Lp(a)] is a highly atherogenic particle in the human plasma (1, 2, 3, 4, 5, 6). Up to 90% of the variance in Lp(a) concentrations is determined by the *LPA* gene (1). *LPA* encodes for apolipoprotein(a) [apo(a)], which consists of 10 different kringle IV domains (KIV-1 to KIV-10). Notably, the KIV-2 is encoded by a 5.6 kb large coding copy number variation (KIV-2 repeat), which leads to >40 apo(a) protein isoforms in the population (1). The apo(a) isoforms are inversely correlated with Lp(a) plasma concentrations, with up to 10 times higher median Lp(a) concentrations observed in low molecular weight (LMW, 10–22 KIV repeats) isoform carriers than in high molecular weight (HMW, >22 KIV repeats) isoform carriers (1, 7). In heterozygous individuals, the smaller isoforms commonly determine the Lp(a) concentrations (albeit not always) (8). However, if stratifying individuals by their isoform, the individual Lp(a) concentrations within each group can nonetheless vary by up to 200-fold (9). This huge variance within each isoform size group cannot be explained by the sole isoform size (9), nor by the second isoform present in heterozygotes (10). For examples, while LMW isoforms are associated with up to 10 times higher median Lp(a) than HMW isoforms, at individual level, 30% of all LMW isoform carriers present Lp(a) <30 mg/dl (11). Such unexpected Lp(a) trait patterns have been termed recently *discordant phenotypes* (12) and have been shown to result from multiple functional variants modifying and partially overruling the impact of the isoform sizes (12, 13). Thus, a complete understanding of the complex Lp(a) trait requires a thorough understanding of the many functional SNPs that govern the trait additionally to the isoforms (recently reviewed in (7)). This promises to allow highly accurate prediction of Lp(a) concentrations from genetic data (13, 14, 15, 16).

Several studies searched for causal genetic determinants of these peculiar phenotypes but causal variants have remained elusive until recently (12, 13, 17, 18, 19, 20). The search for genetic variants regulating Lp(a) has been complicated by the fact that multiple genetic factors cooperate in a nonlinear manner in determining Lp(a) concentrations, such as isoform size, SNPs, and ancestry (7, 21). Interactions between these factors can mask true effects, and many SNPs indeed occur only in narrow isoform ranges (7).

Nonetheless, some early studies succeeded in identifying markers for these discordant phenotypes. One of these are long alleles of a short tandem repeat (STR) located 1.3 kb upstream from the first exon of *LPA* (hg19 chr6:161,086,617–161,086,663; hg38 chr6:160,665,587–160,665,631) in the *LPA* promoter (22, 23, 24, 25, 26). This STR has been named pentanucleotide repeat (PNR) and presents between about 5 and 12 repeats of a TTTTA motif (22, 27). Early studies showed that especially the alleles PNR10 (10 repeat units) and PNR11 (11 repeat units) are associated with shorter isoforms (<24 KIV, i.e., <15 KIV-2 units) expressing extraordinarily low Lp(a) concentrations (23, 24, 28). An in vitro study (22) reported a lower transcriptional activity of promoter fragments carrying allele PNR9 to PNR12, compared to the most common allele in the population (PNR8), but this effect could not be replicated by subsequent studies (23, 29, 30). Surprisingly, epidemiological studies nevertheless observed a clear association between the PNR and Lp(a) plasma concentrations (23, 24, 26, 28, 31, 32, 33, 34). This is intriguing and suggests a linkage disequilibrium (LD) with a yet unknown functional variant causing discordant phenotypes. For instance, an LD with the *LPA* KIV-8 variant rs41272110 (p.Thr1399Pro, traditionally known as Thr3888Pro) has been proposed as a mechanistic basis for the low Lp(a) concentrations observed in PNR10 allele carriers (24, 26). However, no biochemical Lp(a)-lowering mechanism is known for rs41272110 and the effect of rs41272110 was not consistent across different PNR alleles (26).

Recently we have identified a strong LD between rs41272110 and the frequent, strongly Lp(a) and coronary artery disease (CAD) risk–lowering splice variant 4925G>A in the KIV-2 region (35). We showed that the effect of rs41272110 on Lp(a) and on CAD risk depends on the KIV-2 4925G>A and that the Lp(a)-lowering effect previously attributed to rs41272110 is indeed caused by this splice variant (35). The reported LD between rs41272110 and PNR10 (24) led us to the hypothesis that the association of long PNR alleles with low Lp(a) concentrations also could be mediated by KIV-2 4925G>A. This would finally provide a solution to the long-standing riddle about the association of this nonfunctional promoter STR with lower Lp(a) concentrations.

## Material and methods

### Study populations

The population-based study Cooperative Health Research in the Region of Augsburg (KORA) F4 (36) is a follow-up study of the previous KORA S4 study and includes 3,080 participants with German nationality aged 25–74 years, being recruited from 2006 to 2008. The study was performed in accordance with the Declaration of Helsinki and was approved by the Ethics Committee of the Bavarian Medical Association (Bayrische Landesärztekammer). Lp(a) concentrations, apo(a) isoforms, and variant data (rs41272110, KIV-2 4925G>A, and PNR allele) were available for 2,858 participants and were measured at the Institute of Genetic Epidemiology at the Medical University of Innsbruck. Study population characteristics and carrier frequencies of the variants are given in Table 1.

**Table 1:** Study population characteristics

Characteristics	KORA F4
N	2,858
Age, y	56 (44, 67)
Female sex	1,482 (51.8%)
Lp(a), mg/dl	11.7 (5.2, 30.3)
Total cholesterol, mg/dl	214 (188, 240)
HDL-C, mg/dl	54 (45, 65)
LDL-C, mg/dl	134 (111, 158)
Triglycerides, mg/dl	105 (72, 151)
Type 2 diabetes mellitus	198 (6.9%)
4925G>A carrier	634 (22.2%)
MAF rs41272110 (genotype counts TT/TC/CC)	14.2% (3,424/1,063/88)

HDL-C, HDL-cholesterol; KORA, Cooperative Health Research in the Region of Augsburg; LDL-C, LDL-cholesterol; Lp(a), lipoprotein(a); PNR, pentanucleotide repeat.

PNR allele frequencies are given in Fig. 2. Values are provided as median (interquartile range) unless specified differently.

### Lp(a) phenotyping

Lp(a) concentrations were determined in mg/dl by ELISA with a polyclonal affinity-purified rabbit anti-human apo(a) antibody for coating and a horseradish peroxidase–conjugated monoclonal anti-apo(a) antibody 1A2 (37) for detection (38, 39). Absorbance was assessed on two dilutions (1:150 and 1:1,500), and measurements were applied to a 7-point standard curve.

Apo(a) isoforms were detected by Western blotting (38, 39). One hundred fifty nanograms of Lp(a) and a size standard containing apo(a) isoform 13, 19, 23, 27, and 35 KIV repeats (validated by fiber-FISH (40)) were separated on a 1.46% agarose gel with 0.08% SDS for 18 h at 0.04 A constant current. Semidry electroblotting was performed. The membrane was blocked with 1% BSA, 85 mM NaCl, 10 mM TRIS, and 0.2% Triton X-100 for 30 min at 37°C and incubated with horseradish peroxidase–conjugated 1A2 antibody. ECL substrate (WesternBright Chemilumineszenz Spray, Biozym, Vienna, AT) was applied, and signals were recorded on autoradiography films (Amersham Hyperfilm™ ECL™, GE Healthcare, Chicago, IL).

ELISA and Western blot data were available from previous projects. All apo(a) Western blots have been evaluated by the same experienced researcher. Detailed protocols for both methods have been published in (38).

### Variant typing

In KORA F4 KIV-2, 4925G>A carrier status and PNR genotype were assessed in earlier projects (12, 18, 41). In brief, 4925G>A had been typed using a commercial castPCR assay (ThermoFisher Scientific), which provides positive or negative carrier status (12, 18). The PNR repeats were determined by fluorescent fragment analysis on an ABI 3730s Genetic Analyzer with POP-7 polymer (Applied Biosystems) and assessed using GeneMapper Software version 4.1 (Applied Biosystems) (41). For each heterozygous individual, the shorter PNR allele was assigned to the variable *PNR allele 1* and the longer to the variable *PNR allele 2*. KORA F4 was genotyped using the Affymetrix Axiom. rs10455872 was present directly on the genotyping microarray. rs41272110 and rs3798220 were imputed against the Haplotype Reference Consortium (42) using the Michigan Imputation Server (43) (imputation quality: *R*^2^ = 0.9587 and *R*^2^ = 0.9418).

Investigations on different ancestries were performed in 2,504 high-coverage whole-genome sequencing samples from the 1000 Genomes (1000G) Project (44). Information on the variant calling in 1000G is given in Supplementary Methods.

### Statistical analysis

The variant 4925G>A is located in the repetitive KIV-2 region. Therefore, PCR assays do not differentiate whether a signal showing multiple 4925G>A occurrences stems from multiple mutated KIV-2 repeats on the same chromosome or on different chromosomes. Thus, the variant is reported as positive or negative carrier status (12, 17, 35). LD between the KIV-2 4925G>A and the alleles PNR10 and PNR11 was calculated using the R package *genetics* (https://CRAN.R-project.org/package=genetics). Therefore, as in previous investigations (12, 17), the carrier status was coded as heterozygous.

As PNR alleles with 11 repeats were observed only on the longer PNR allele (PNR allele 2), regression analyses were performed on the longer PNR allele, using PNR8 as a reference allele. To account for the strongly skewed distribution of the Lp(a) trait, the association between variants and Lp(a) concentrations was tested using quantile regression, modeling the conditional median, adjusted for age and sex (model 1) as well as the apo(a) isoform (model 2), as implemented in the R package *quantreg* (45). Sensitivity analysis was performed by adjusting the main regression model additionally for the shorter PNR allele (PNR allele 1). For individuals showing two apo(a) isoforms in the Western blot, the smaller isoform was used for isoform adjustment and for isoform grouping as this is commonly the dominant isoform (8). The only one apo(a) isoform present on the Western blot was used for true homozygous individuals and individuals with only one isoform expressed. For all analyses, R version 4.0.1 was used.

## Results

**Fig. 1:**
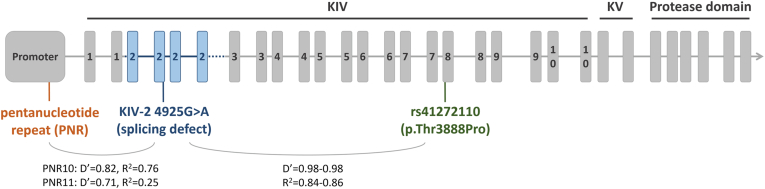
*LPA* gene structure with the LD statistics between the variants discussed.

Previous studies suggested an LD between alleles PNR10 and PNR11 and rs41272110 (24, 26). We have shown recently that the individual effect of rs41272110 on Lp(a) depends on the KIV-2 4925G>A carrier status and that the Lp(a) decrease previously attributed to rs41272110 is produced by a partial LD with 4925G>A (35). Given the occurrence of the alleles PNR10 and PNR11 in the same isoform range as 4925G>A and rs41272110, we hypothesized that a similar mechanism might also cause the association of PNR10 and PNR11 with low Lp(a) plasma concentrations (Fig. 1).

**Fig. 2:**
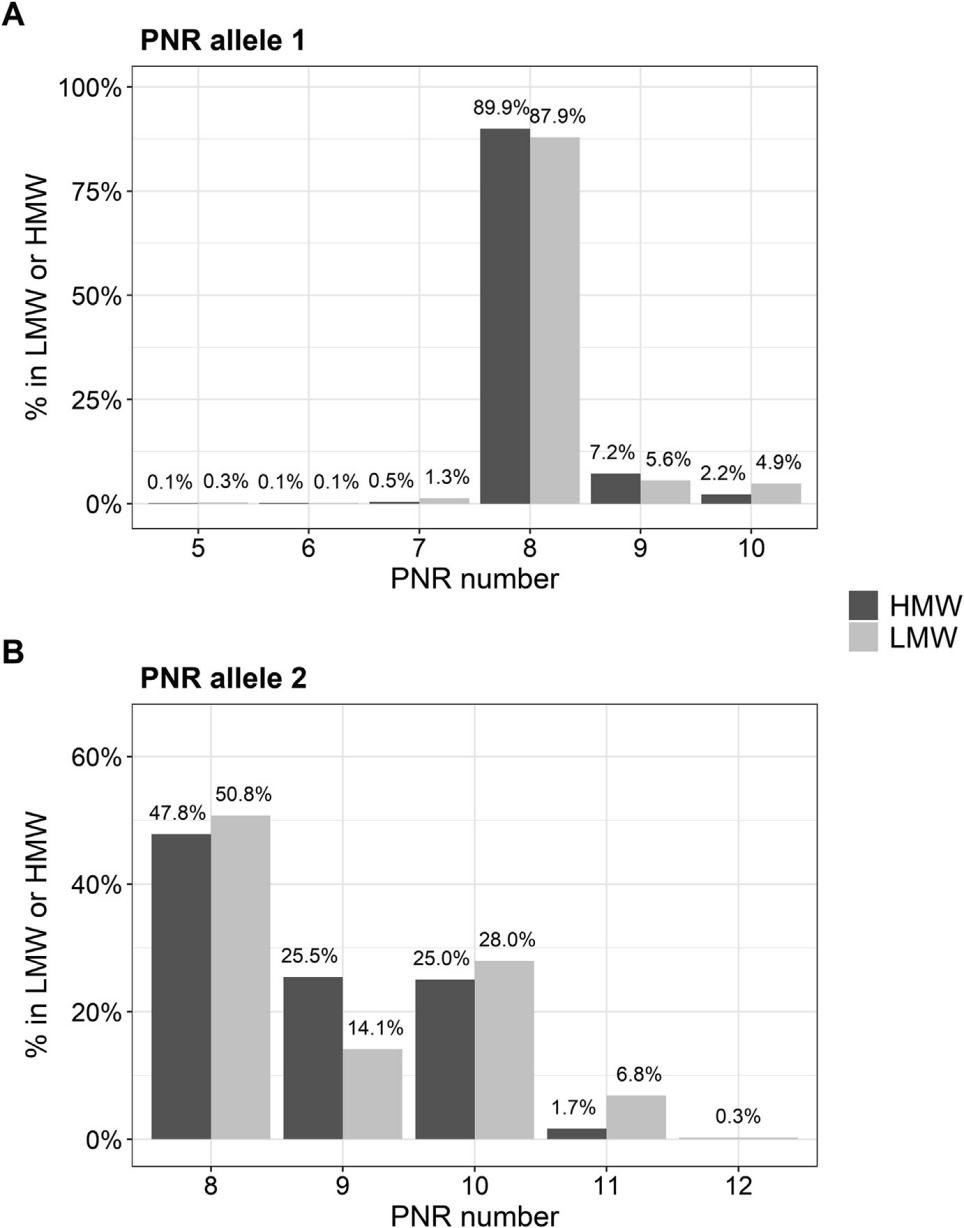
Distribution of the short and the long PNR allele (panel A: PNR allele 1, panel B: PNR allele 2) in the KORA F4 population, stratified by HMW and LMW carriers. Percentage is given per total individuals in LMW or HMW group.

**Fig. 3:**
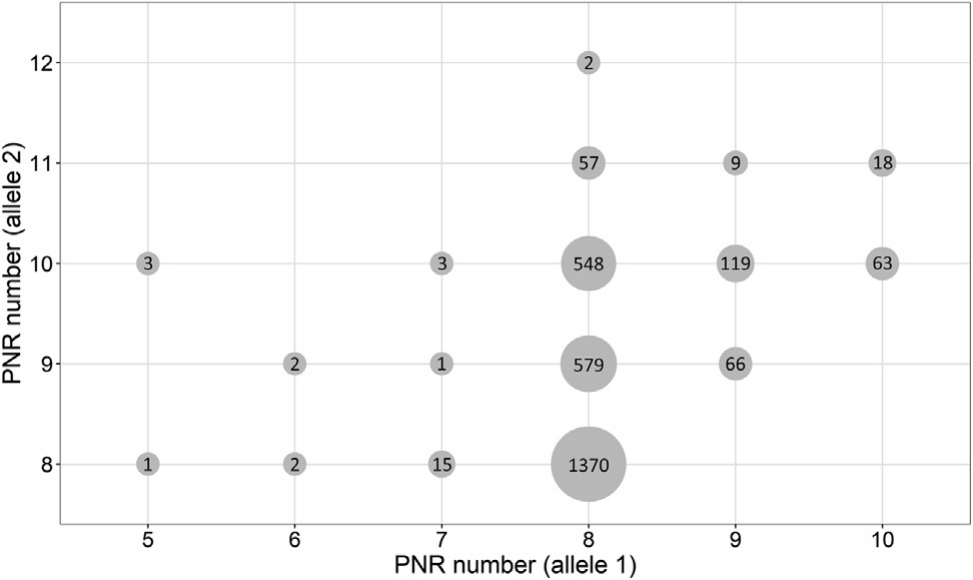
PNR genotypes (PNR allele combinations) in the KORA F4 population (n = 2,858). Numbers in dots represent the individuals showing the PNR genotype resulting from the individual PNR allele 1 and PNR allele 2. Empty line intersections correspond to no carriers.

We observed eight different PNR alleles with 5–12 repeats in the analyzed European population sample. A range of 5–10 repeats was observed in the shorter PNR allele 1 and 8–12 repeats in the longer PNR allele 2. The frequency of the repeat numbers of both PNR alleles stratified by LMW and HMW isoforms is shown in Fig. 2, and the resulting PNR genotypes are shown in Fig. 3. The most frequent genotypes were PNR8/PNR8 (47.9%), PNR8/PNR9 (20.3%), and PNR8/PNR10 (19.2%). As the allele PNR11 was present only on PNR allele 2, all analyses were based on PNR allele 2. The PNR8 allele was the most frequent allele and used as a reference allele for all analyses.

In line with previous findings (23, 24, 25, 26), in an age-, sex-, and isoform-adjusted quantile regression model, both the PNR10 and the PNR11 allele were associated with significantly lower Lp(a) plasma concentrations (PNR10: β = −14.4 mg/dl, *P* = 3.33e-84; PNR11: β = −17.2 mg/dl, *P* = 4.01e-29; confidence intervals for all regression models are shown in the respective tables; Table 2). Of note, this effect was noticeable only after isoform adjustment or stratification. In a non-isoform-adjusted model, the effect was very small in the total population (PNR10: β = −3.1 mg/dl, *P* = 3.86E-05; PNR11: β = −2.2 mg/dl, *P* = 0.0604) but was enhanced by one order of magnitude when the analysis was restricted to LMW samples (PNR10: β = −24.9 mg/dl, *P* = 1.03E-16; PNR11: β = −40.2 mg/dl, *P* = 1.85E-11; Table 2). Conversely, no relevant effects were observed in the HMW groups. Of note, also a minor association of PNR9 with lower Lp(a) was observed in the total population. Unlike PNR10 and PNR11, the overall effect was not modified by isoform adjustment, and despite a similar number of cases as for PNR10 and PNR11, it was no more significant when stratifying by LMW/HMW (Table 2). PNR12 was too rare for meaningful analysis (n = 2). Additional adjustment for the other PNR allele present (PNR allele 1) did not modify the results (supplemental Table S1) and was not applied to further regression models. PNR5 to PNR7 were observed only on the shorter PNR allele (PNR allele 1). PNR5 (n = 4) and PNR6 (n = 4) were too rare for analysis, and no association with Lp(a) was observed in carriers of PNR7 (n = 19).

**Table 2:** Quantile regression analysis of PNR allele 2 on Lp(a) concentration

Group	Variant	Carrier [N]	Median Lp(a) Concentration (IQR)	Model 1: Adjusted for Age and Sex			Model 2: Adjusted for Age, Sex, and the Smaller apo(a) Isoform		
				β	95% CI	*P*	β	95% CI	*P*
All	PNR8	1,388	13.4 (4.8, 39.0)	Reference					
	PNR9	648	11.1 (4.6, 23.7)	−2.07	−3.95, −0.19	0.0313	−2.78	−3.72, −1.83	1.05E-08
	PNR10	736	10.4 (6.2, 23.1)	−3.10	−4.57, −1.63	3.86E-05	−14.43	−15.84, −13.02	3.33E-84
	PNR11	84	10.9 (5.5, 19.6)	−2.23	−4.57, 0.10	0.0604	−17.21	−20.19, −14.23	4.01E-29
	PNR12	2	NA	NA	NA	NA	NA	NA	NA
LMW	PNR8	356	53.2 (40.3, 74.4)	Reference					
	PNR9	99	49.2 (31.4, 64.6)	−5.61	−13.08, 1.86	0.0719	−4.72	−11.98, 2.53	0.2027
	PNR10	196	28.2 (10.8, 54.5)	−24.92	−30.77, −19.07	1.03E-16	−24.16	−29.94, −18.39	1.11E-15
	PNR11	48	12.5 (5.7, 23.9)	−40.20	−50.30, −30.11	1.85E-11	−39.50	−49.31, −29.69	1.13E-14
	PNR12	2	NA	NA	NA	NA	NA	NA	NA
HMW	PNR8	1,032	8.7 (3.6, 18.3)	Reference					
	PNR9	549	9.4 (3.8, 17.3)	0.54	−0.88, 1.96	0.4539	0.55	−0.71, 1.81	0.3895
	PNR10	540	8.6 (5.3, 14.5)	−0.24	−1.32, 0.84	0.6630	−2.88	−3.85, −1.91	7.13E-09
	PNR11	36	9.2 (4.2, 17.3)	0.91	−2.83, 4.65	0.6344	−0.69	−3.62, 2.24	0.6456
	PNR12	0	NA	NA	NA	NA	NA	NA	NA

HMW, high molecular weight; LMW, low molecular weight; Lp(a), lipoprotein(a); PNR, pentanucleotide repeat.

In all models PNR8 allele was used as reference allele. Median Lp(a) concentration and effect are given in mg/dl.

In summary, the regression models revealed a strong isoform-specific effect of PNR10 and PNR11 in the total group, which stems from a very strong Lp(a)-lowering effect in the LMW group. Given the aforementioned coincidence of the strongly Lp(a)-lowering splice site mutation in the KIV-2 4925G>A in the same isoform range (12), we hypothesized a role of this mutation in establishing the observed Lp(a)-lowering effect of the PNR.

**Fig. 4:**
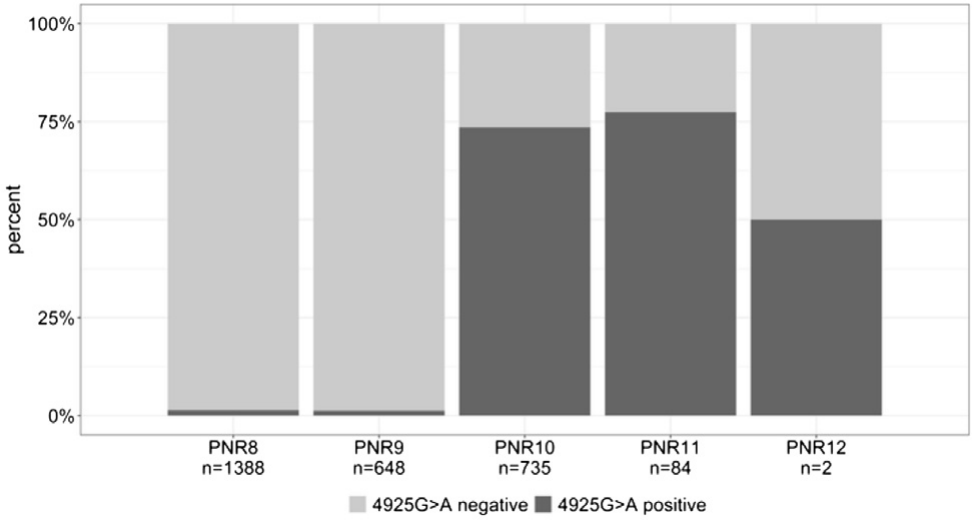
Carrier frequency of KIV-2 4925G>A in each PNR allele. A large fraction of PNR10 and PNR11 carriers carries also KIV-2 4925G>A (depicted in dark gray), leading to lower Lp(a) concentration. Numbers of individuals carrying 4925G>A separated for PNR allele: PNR8: n = 19; PNR9: n = 8; PNR10: n = 541, PNR11: n = 65, PNR12: n = 1.

Indeed, Fig. 4 shows a very high 4925G>A carrier frequency in individuals with the PNR alleles PNR10 (73.6%) and PNR11 (77.4%), compared to a carrier frequency of 22.2% in the entire population. Conversely, 4925G>A carrier frequency was very low in individuals with the PNR alleles PNR8 (1.4%) and PNR9 (1.2%). Accordingly, a high Lewontins D’ (and high to moderate *R*^2^) was observed between the two PNR alleles and 4925G>A (PNR10: D’ = 0.82, *R*^2^ = 0.76; PNR11: D’ = 0.71, *R*^2^ = 0.25). High D′ suggests a low likelihood of recombination between two loci and, accordingly, that both SNPs are most often inherited together. We have shown before that effects of correlated *LPA* SNPs on Lp(a) can be mediated also via the D′ structure instead of the *R*^2^ structure (18) (reviewed in (7)).

**Fig. 5:**
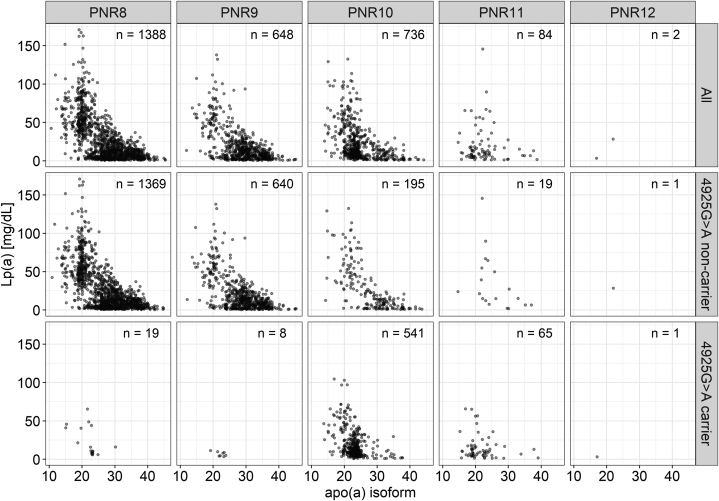
Distribution of the PNR alleles across the different apo(a) isoform sizes and Lp(a) concentration. The first row shows the total population, while the second and third rows show noncarriers and carriers of 4925G>A, respectively. A large proportion of the PNR10 and PNR11 carriers with low Lp(a) concentrations carry KIV-2 4925G>A as well. The remaining variance in Lp(a) concentration in carriers of 4925G>A results mostly from the second unaffected allele.

Figure 5 shows the distribution of the PNR alleles across the different apo(a) isoform sizes and Lp(a) concentrations. It shows that a considerable number of PNR10 and PNR11 carriers with low Lp(a) concentrations in the isoforms range <25 KIV indeed carry 4925G>A as well. Accordingly, adjustment for 4925G>A completely abolishes the effect of PNR10 and PNR11 alleles on Lp(a) concentration in both models (age- and sex-adjusted model: PNR10: β = +1.03, *P* = 0.13; PNR11: β = +1.84, *P* = 0.22; isoform-adjusted model: PNR10: β = +0.44, *P* = 0.69; PNR11: β = −1.52, *P* = 0.51; Table 3).

**Table 3:** Quantile regression analysis of PNR allele 2 on Lp(a) concentration adjusted for 4925G>A (joint model)

Group	Variant	Carrier [N]	Median Lp(a) Concentration (IQR)	Model 1: Adjusted for Age and Sex			Model 2: Adjusted for Age, Sex, and the Smaller apo(a) Isoform		
				β	95% CI	*P*	β	95% CI	*P*
All	PNR8	1,388	13.4 (4.8, 39.0)	Reference					
	PNR9	648	11.1 (4.6, 23.7)	−1.98	−3.68, −0.28	0.0229	−2.97	−4.1, −1.85	2.32E-07
	PNR10	736	10.4 (6.2, 23.1)	1.03	−0.29, 2.34	0.1270	0.44	−1.73, 2.60	0.6927
	PNR11	84	10.9 (5.5, 19.6)	1.84	−1.07, 4.74	0.2154	−1.52	−6.05, 3.00	0.5102
	PNR12	2	NA	NA	NA	NA	NA	NA	NA
	4925G>A	634	9.7 (6.3, 17.6)	−4.73	−6.16, −3.31	8.65E-11	−20.98	−23.46, −18.51	2.03E-59
LMW	PNR8	356	53.2 (40.3, 74.4)	Reference					
	PNR9	99	49.2 (31.4, 64.6)	−5.58	−11.96, 0.80	0.0871	−5.04	−11.42, 1.34	0.1220
	PNR10	196	28.2 (10.8, 54.5)	−2.84	−10.25, 4.56	0.4520	1.90	−5.56, 9.37	0.6174
	PNR11	48	12.5 (5.7, 23.9)	−10.82	−21.62, −0.01	0.0502	−8.80	−19.61, 2.01	0.1111
	PNR12	2	NA	NA	NA	NA	NA	NA	NA
	4925G>A	196	15.1 (8.4, 39.6)	−32.85	−23.46, −18.51	1.90E-16	−35.02	−42.65, −27.38	2.31E-18
HMW	PNR8	1,032	8.7 (3.6, 18.3)	Reference					
	PNR9	549	9.4 (3.8, 17.3)	0.47	−0.76, 1.71	0.4539	0.43	−0.75, 1.62	0.4735
	PNR10	540	8.6 (5.3, 14.5)	0.32	−1.05, 1.70	0.6434	0.95	−1.09, 2.98	0.3625
	PNR11	36	9.2 (4.2, 17.3)	1.58	−1.42, 4.58	0.3030	2.40	−1.35, 6.15	0.2098
	PNR12	0	NA	NA	NA	NA	NA	NA	NA
	4925G>A	438	8.6 (6.0, 13.2)	−0.65	−1.82, 5.53	0.2812	−5.55	−7.82, −3.28	1.71E-06

HMW, high molecular weight; LMW, low molecular weight; Lp(a), lipoprotein(a); PNR, pentanucleotide repeat.

In all models PNR8 allele was used as reference allele. Median Lp(a) concentration and effect are given in mg/dl.

We recently reported also a strong LD between 4925G>A and the well-known missense *LPA* variant Thr3888Pro (p.Thr1399Pro, rs41272110) (35). As others had suggested that the effect of the PNR on Lp(a) might derive from rs412722110 (24, 26), we investigated whether the effect of PNR10 and PNR11 allele could indeed be caused by an LD with rs41272110 instead of an LD with 4925G>A. In contrast to the regression models including 4925G>A, inclusion of rs41272110 into the regression models did not abolish the association of PNR10 and PNR11 alleles with low Lp(a) (isoform-adjusted model: PNR10: β = −8.74, *P* = 3.89e-16; PNR11: β = −11.52, *P* = 5.71e-13; supplemental Table S2). Conversely, adding subsequently also KIV-2 4925G>A to the regression model with rs41272110 showed a significant Lp(a)-decreasing effect of 4925G>A (β = −25.42, *P* = 3.05E-40), a significant Lp(a) increase in carriers of rs41272110 (β = +4.49, *P* = 0.0018), which is in line with what we have shown previously (35), and confirm no effect of the PNR alleles PNR10 or PNR11 (PNR10: β = +0.24, *P* = 0.82; PNR11: β = −1.84, *P* = 0.25; Table 4). This further supports that the effect of the PNR alleles PNR10 and PNR11 allele on Lp(a) is, indeed, caused by KIV-2 4925G>A and not by rs41272110. Additionally, we also stratified our data for rs10455872 and rs3798220 (known proxy SNPs for LMW isoforms (46) and high Lp(a)) to investigate the interplay between these two variants and KIV-2 4925G>A, but no overlap between these variants was evident (supplemental Figs. S1–S3 and supplemental Table S3).

**Table 4:** Quantile regression analysis of PNR allele 2 on Lp(a) concentration in joint models including rs41272110 and 4925G>A

Group	Variant	Carrier [N]	Median Lp(a) Concentration (IQR)	Model 1: Adjusted for Age and Sex			Model 2: Adjusted for Age, Sex, and the Smaller apo(a) Isoform		
				β	95% CI	*P*	β	95% CI	*P*
All	PNR8	1,388	13.4 (4.8, 39.0)	Reference					
	PNR9	648	11.1 (4.6, 23.7)	−2.68	−4.42, −0.94	0.0025	−3.37	−4.50, −2.24	5.66E-09
	PNR10	736	10.4 (6.2, 23.1)	0.78	−1.32, 2.89	0.4660	0.24	−1.80, 2.28	0.8154
	PNR11	84	10.9 (5.5, 19.6)	0.64	−2.38, 3.65	0.6783	−1.84	−4.96, 1.28	0.2473
	PNR12	2	NA	NA	NA	NA	NA	NA	NA
	rs41272110	744	11.1 (6.8, 25.0)	10.18	6.04, 14.32	1.49E-06	4.49	1.68, 7.31	0.0018
	4925G>A	634	9.7 (6.3, 17.6)	−15.29	−19.64, −10.94	6.78E-12	−25.42	−29.11, −21.73	3.05E-40
LMW	PNR8	356	53.2 (40.3, 74.4)	Reference					
	PNR9	99	49.2 (31.4, 64.6)	−4.78	−11.13, 1.58	0.1415	−5.37	−12.74, 2.01	0.1542
	PNR10	196	28.2 (10.8, 54.5)	−3.83	−12.52, 4.86	0.3881	−2.13	−12.22, 7.96	0.6792
	PNR11	48	12.5 (5.7, 23.9)	−11.99	−23.84, −0.14	0.0477	−13.09	−26.84, 0.65	0.0624
	PNR12	2	NA	NA	NA	NA	NA	NA	NA
	rs41272110	239	21.1 (9.3, 51.4)	4.48	−3.63, 12.59	0.2790	6.18	−3.28, 15.64	0.2008
	4925G>A	196	15.1 (8.4, 39.6)	−37.45	−46.35, −28.55	8.08E-16	−38.13	−48.48, −27.79	1.30E-12
HMW	PNR8	1,032	8.7 (3.6, 18.3)	Reference					
	PNR9	549	9.4 (3.8, 17.3)	−0.18	−1.46, 1.10	0.7849	0.00	−1.31, 1.30	0.9951
	PNR10	540	8.6 (5.3, 14.5)	0.28	−1.45, 2.01	0.7475	0.67	−0.56, 1.90	0.4800
	PNR11	36	9.2 (4.2, 17.3)	1.95	−1.97, 5.88	0.3295	2.61	−0.43, 5.64	0.1018
	PNR12	0	NA	NA	NA	NA	NA	NA	NA
	rs41272110	505	9.4 (6.2, 16.5)	7.52	6.36, 8.69	1.27E-35	−11.01	−13.66, −8.36	8.50E-09
	4925G>A	438	8.6 (6.0, 13.2)	−8.33	−10.30, −6.35	2.41E-16	−0.47	−0.57, −0.37	6.01E-16

HMW, high molecular weight; LMW, low molecular weight; Lp(a), lipoprotein(a); PNR, pentanucleotide repeat.

The model uses PNR8 allele as reference allele. Median Lp(a) concentration and effect are given in mg/dl.

As Lp(a) levels and also carrier frequencies of variants in the *LPA* gene differ across populations, we additionally investigated the LD between the PNR alleles and KIV-2 4925G>A on the 1000G Project whole-genome sequencing dataset (n = 2,504). This showed different carrier frequencies of KIV-2 4925G>A and of the PNR alleles across the different populations (supplemental Figs. S4 and S5; supplemental Table S4), as previously shown in (12, 23, 29, 31). Interestingly, we detected PNR4 alleles in 1.8% (n = 9) of the East Asian population (supplemental Fig. S4). Alleles with such low repeat numbers had been observed in East Asians also in another earlier study (47). LD statistics for the different populations are reported in supplemental Table S4. In Non-Finnish Europeans, the LD between PNR10 and PNR11 and 4925G>A (PNR10: D’ = 0.84, R^2^ = 0.76; PNR11: D’ = 0.71, R^2^ = 0.26) was indeed very similar to our observations in KORA F4 (PNR10: D’ = 0.82, R^2^ = 0.76; PNR11: D’ = 0.71, R^2^ = 0.25). Globally, the LD between KIV-2 4925G>A and the PNR10 and PNR11 alleles was highest in Europeans and South Asians (supplemental Table S5).

## Discussion

Several studies investigated the role of the *LPA* promoter PNR in the regulation of plasma Lp(a) concentrations (22, 23, 24, 27, 28, 29, 30, 31, 33, 34). Different PNR alleles reproducibly presented a strong and highly significant association with Lp(a) concentrations (24, 26, 28, 31, 32, 33, 34), despite the PNR has no direct impact on promoter activity (23, 29, 30). The relevance of the PNR and the reason for its association with Lp(a) concentrations is, therefore, still unknown (7). We robustly replicated the association of long PNR alleles with strongly lowered Lp(a), especially in LMW isoforms, and found that the association with low Lp(a) is indeed caused by a strong LD between PNR10 and PNR11 and the splice site variant 4925G>A (Fig. 1). KIV-2 4925G>A is located within the repetitive KIV-2 region. The variant reduces splicing efficiency by activating a cryptic intronic splice site (12). This results in an intron retention creating a premature stop codon and leads to reduced protein production (12), which in turn is associated with decreased CAD risk (35, 48). LMW and HMW carriers present rather different median Lp(a) concentrations. Carriers of KIV-2 4925G>A are predominantly found in LMW and shorter HMW isoform sizes (23–25 KIV repeats) (12), and thus, the beta estimates for the longer PNR alleles are larger in the LMW than in the HMW isoform groups. The effect of 4925G>A and of the large PNR alleles is large in the range of 23–25 KIV (KIV-2 4925G>A: β = −15.48 mg/dl; PNR10: β = −12.43 mg/dl; PNR11: β = −9.64 mg/dl), but is diluted when analyzing the whole HMW group.

Prins *et al.* (24) described an LD between the PNR10 allele and a substitution from threonine to proline in KIV-8 (rs41272110; p.Thr1399Pro, traditionally named Thr3888Pro). Other studies report an LD of different PNR alleles also with other coding variants, predominantly located in the region of KIV-8 to KIV-10 (24, 25, 26), but no variant markedly accounted for the effect of any PNR allele (26). This supports the hypothesis that the association of the PNR with Lp(a) is mediated by a to date unidentified functional variant (23). Recently, we have shown that rs41272110 and the strongly Lp(a)-lowering KIV-2 splice site variant 4925G>A are in strong LD and that the Lp(a)-lowering effect previously attributed to rs41272110 is indeed caused by 4925G>A (35). This remained undetected until now because 4925G>A was hidden in the complex KIV-2 repeat region and became accessible to genetic-epidemiological studies only recently (12, 49). Also the PNR has been neglected by recent large-scale genetic studies because, unlike SNPs, STRs cannot be inexpensively determined in large populations in an automated manner. Thus, both polymorphisms had escaped detailed attention so far. Having both variants available from previous efforts in a large population-based study (n = 2,858) enabled us to investigate the interplay of these two elusive variants.

Individuals carrying both the PNR10 allele and KIV-2 4925G>A encompass a very large proportion of the PNR10 allele carriers with low Lp(a) concentrations (Fig. 5). Some small remaining clusters of individuals with low Lp(a) despite small isoforms might be explained by other Lp(a) decreasing variants. Indeed, Mukamel *et al.* (13) have recently defined 23 mostly loss-of-function variants that are associated with significantly lower Lp(a) across the complete minor allele frequency spectrum. Additionally, several studies describe SNP haplotypes within same-sized isoforms (7). Said *et al.* (20) for example describe an interplay between a rare missense variant and rs10455872 that lowers the median Lp(a) concentration in carriers of both variants. Conversely, we observed that carriers of rs10455872 and rs3798220 are located on a different haplotype than KIV-2 4925G>A, despite being both predominantly found in LMW isoforms.

Interestingly, Trommsdorff *et al.* (29) showed that there is no correlation between PNR and lower Lp(a) levels in African American individuals. Our data from the 1000G Project dataset suggest that this is due to the low carrier frequency of KIV-2 4925G>A (1.8%) in African individuals. The same observation could be translated also to the East Asian population. Due to this low frequency, the relevance of the splicing variant at an individual level in these populations is lower than in Europeans. However, such frequency differences of mainly Lp(a)-lowering variants may cooperate in establishing the observed differences in Lp(a) between ancestries. Mukamel *et al.* (13) indicated that the paucity of 4925G>A carriers in African populations possibly explains the higher median Lp(a) concentration in this population than in European individuals. Investigations on the LD between the apo(a) isoform and the PNR genotype in different populations are sparse. Various studies report data, but with a high heterogeneity between the studies (23, 29, 50).

Previous authors proposed rs41272110 as another possible causal SNP behind the association of PNR10 and PNR11 with low Lp(a). Very recently, we reported a high LD (*R*^2^ = 0.84–0.86; D’ = 0.98–0.99) between KIV-2 4925G>A and rs41272110 and showed that the decreasing effect in carriers of rs41272110 is caused by the splice variant. Indeed, we could show that addition of rs41272110 to the regression model does not abolish the association, while additional inclusion of 4925G>A does abolish the association with low Lp(a) of both the PNR and of rs41272110 (35). As shown previously, adjustment for 4925G>A also reveals a hidden Lp(a)-increasing effect of rs41272110 (35). This underscores the frequent splice site variant KIV-2 4925G>A as the major contributor to the association of the PNR with Lp(a).

The huge variance observed in the Lp(a) trait and even within each isoform group has puzzled Lp(a) research for a long time as causal variants have been elusive. Only very recently, multiple variants were identified (12, 13, 17, 18, 19, 48) and shed light on the genetic basis of this variance. Our study adds to these recent works and clarifies the causal basis of a further *LPA* polymorphism that had been linked with discordant phenotypes. It remains to be seen how many independent causal entities exist in the *LPA* gene, but elucidating all of them now appears to come into reach. For example, Mukamel *et al.* (13) proposed a manageable set of 23 variants that, together with the apo(a) isoforms, promise to explain a large portion (>80%) of Lp(a) variance. This puts even accurate genetic prediction of Lp(a) concentrations from genomic data within reach and may allow accurate stratification of individuals even without available Lp(a) measurements. It has been proposed recently that this could be used, for example, to identify individuals who most likely qualify for a given trial or therapy within available cohorts (15). In light of these advancements, elucidation of variants associated with discordant phenotypes has proven very important to complete our understanding of the Lp(a) trait (7, 13). Our work shows that the PNR is not an independent variant and most likely does not need to be taken into account. This might be a reassuring message for the community as microsatellites are still poorly captured by current high-throughput genomic technologies.

### Strengths and limitations

Both the KIV-2 4925G>A splice site variant and the PNR are elusive *LPA* variants that are not commonly available in any large epidemiological study. Encompassing more than 2,800 individuals from a population-based cohort, our study is to the best of our knowledge the currently largest study combining data of the PNR, *LPA* SNPs, KIV-2 SNPs, and Lp(a) concentrations and directly determined apo(a) isoforms. This put us in the unique position to address the interplay of these two discussed variants. We are aware that this study focuses on two specific variants. The functionality of the KIV-2 4925G>A variant has been shown by previous studies (12), and it has been included in a highly curated set of very likely functional *LPA* SNPs (13), but, clearly, we cannot definitely rule out the role of another yet unidentified polymorphism. Our observations highlight the need of scaling up such analyses up to locus- or genome-wide approaches in future studies. We acknowledge that we could not perform analyses at genotype level because the high polymorphic rate of the PNR still results in a lack of power for the single genotypes.

## Conclusion

We found that PNR10 and PNR11 do not contribute to the variance of Lp(a) concentrations. Their apparent effect on Lp(a) is solely mediated by an LD with the KIV-2 splice site mutation 4925G>A. This study highlights the complexity of the genetic regulation of Lp(a) and how the effect of an *LPA* polymorphism on Lp(a) concentration can derive from other, hidden variants in partial LD. Elucidating these complex interactions will bring us closer to a comprehensive understanding of the high variance of the Lp(a) trait and its genetic regulation.

## Data availability

Data from the KORA F4 study were used under license for the respective study and are not publicly available. Data access requires formal application to the steering committees. However, data supporting the findings are available from the corresponding author upon reasonable request and with permission from the respective steering committees. Requests to access the dataset can be made using the digital tool KORA.PASST in accordance with the informed consent given by the study participants (https://www.helmholtz-munich.de/epi/research/cohorts/kora-cohort/datause-and-access-via-korapasst). The 1000G data and all analysis tools are available at the locations provided in the Supplementary Methods section.

## Supplemental data

This article contains supplemental data (12, 44, 51, 52, 53, 54, 55, 56, 57, 58, 59).

## Conflict of interest

F. K. has served on the advisory boards and has received lecture fees from Novartis, Amgen, and Kaneka. S. C., C. L., and L. F. have received lecture fees from Novartis. The other authors disclose no conflicts of interest.
