# Supplementary material for: The kringle IV type 2 domain variant 4925G>A causes the elusive association signal of the LPA pentanucleotide repeat
Source: J Lipid Res. 2022 Oct 26;63(12):100306. doi: 10.1016/j.jlr.2022.100306 (PMC9700027; doi:10.1016/j.jlr.2022.100306)
Supplement: Supplemental Figures S1–S5 and Tables S1–S5 [file mmc1.pdf]

Supplemental materials to

The kringle IV type 2 domain variant 4925G>A causes the elusive association signal of the *LPA* pentanucleotide repeat

Short title: Low Lp(a) in carriers of long PNR is caused by KIV-2 4925G>A

Rebecca Grüneis, MSc[1], Hansi Weissensteiner, PhD[1], Claudia Lamina, PhD[1], Sebastian Schönherr, PhD[1], Lukas Forer, PhD[1], Silvia Di Maio, MSc[1], Gertraud Streiter, BSc[1], Annette Peters, PhD[2,3,4], Christian Gieger, PhD[2,3,5], Florian Kronenberg ,MD[1], Stefan Coassin, PhD[1]

[1] Institute of Genetic Epidemiology, Department of Genetics, Medical University of Innsbruck
[2] German Center for Diabetes Research (DZD), München-Neuherberg, Germany
[3] Institute of Epidemiology, Helmholtz Zentrum München - German Research Center for Environmental Health, Neuherberg, Germany
[4] Department of Epidemiology, Institute for Medical Information Processing, Biometry and Epidemiology, Ludwig-Maximilians-University Munich, Munich, Germany
[5] Research Unit of Molecular Epidemiology, Helmholtz Zentrum München, German Research Center for Environmental Health, Neuherberg, Germany

Supplementary Methods

Analysis of the *LPA* PNR in the 1000 Genomes Project

For the analysis of the PNR in the 1000 Genomes Project (1), we downloaded the STR region at chromosome 6 with SAMtools (2) v1.16 by extending it by 2 kb from start and end of the locus (hg38 chr6:160,663,087-160,667,831) for all 2,504 high coverage WGS samples (3). Subsequently we used GangSTR (4) and HipSTR (5) for the STR analysis. 2,465 samples analysed with HipSTR met the requirement of a coverage of at least 10 reads after removal of duplicates (--min-reads 10). The remaining samples had a coverage of at least >=6x after removal of duplicate reads, with 1 sample failing the analysis. GangSTR (v.2.5.0) was successfully run on all 2,504 samples with adapted parameters --insertsmean and --insertsdev for fragment length mean (mean insert size 424.1) and its standard deviation (mean 84.7) per sample estimated with SAMTools respectively. The resulting VCF files were compared in R with vcfR package (6). A correlation of r=0.949 over all samples for shorter and longer repeat respectively between HipSTR and GangSTR was observed. The values increased to r=0.971 and 0.991 when excluding the 38 low coverage samples in HipSTR for shorter and longer repeat respectively. After manual inspection of differing BAM files in Unipro UGENE (7), we found GangSTR to be more reliable. Thus all subsequent analyses were performed on the GangSTR dataset.

Analysis of the 4925G>A variant in the 1000 Genomes Project

The complete *LPA* locus from the 1000 Genomes Project high coverage data (hg38: chr6:160,531,483-160,666,375) for all 2,504 samples was downloaded with SAMtools. Subsequently the BAM files were analysed by limiting the region to the KIV type-2 locus, remapping the reads to a single copy of the repeat as presented earlier (8). For this purpose, we used a Nextflow-pipeline based on the mutserve variant caller (9). Overall, a mean coverage of 660.6x (SD 79.8, min. 199x, max. 1,626x) was observed over the KIV type-2.

Supplementary Figures

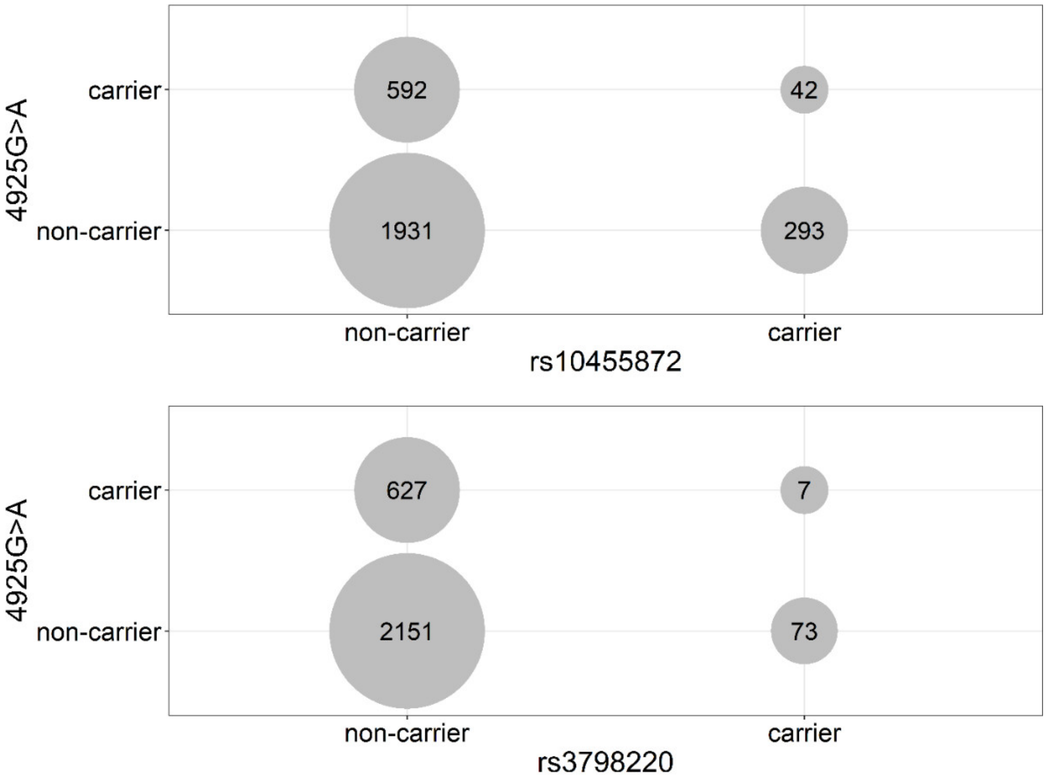

Supplementary Figure I. Cross tables of rs10455872, rs3798220 and KIV-2 4925G>A to investigate the interplay between these variants. Carriers of rs10455872/rs3798220 are defined as carriers of at least one minor allele copy. Rs10455872 and rs3798220 carriers occur both in the LMW range. However, do not significantly overlap with carriers of KIV-2 4925G>A. Median Lp(a) concentrations of the single groups are shown in Supplementary Table III.

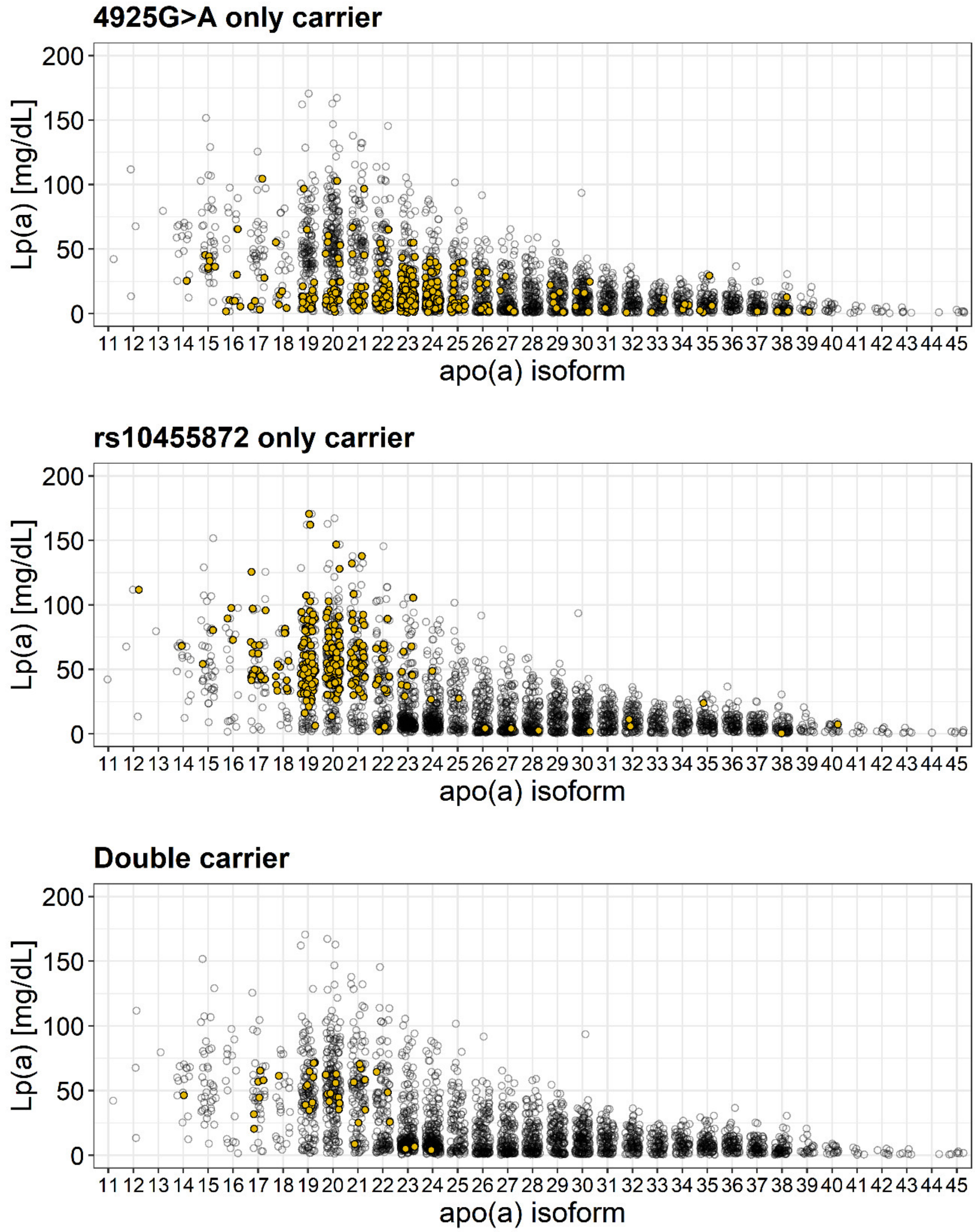

Supplementary Figure II. Lp(a) concentrations of different combinations of KIV-2 4925G>A and rs10455872 distributed over the different isoform sizes. Carriers per plot are highlighted in blue.

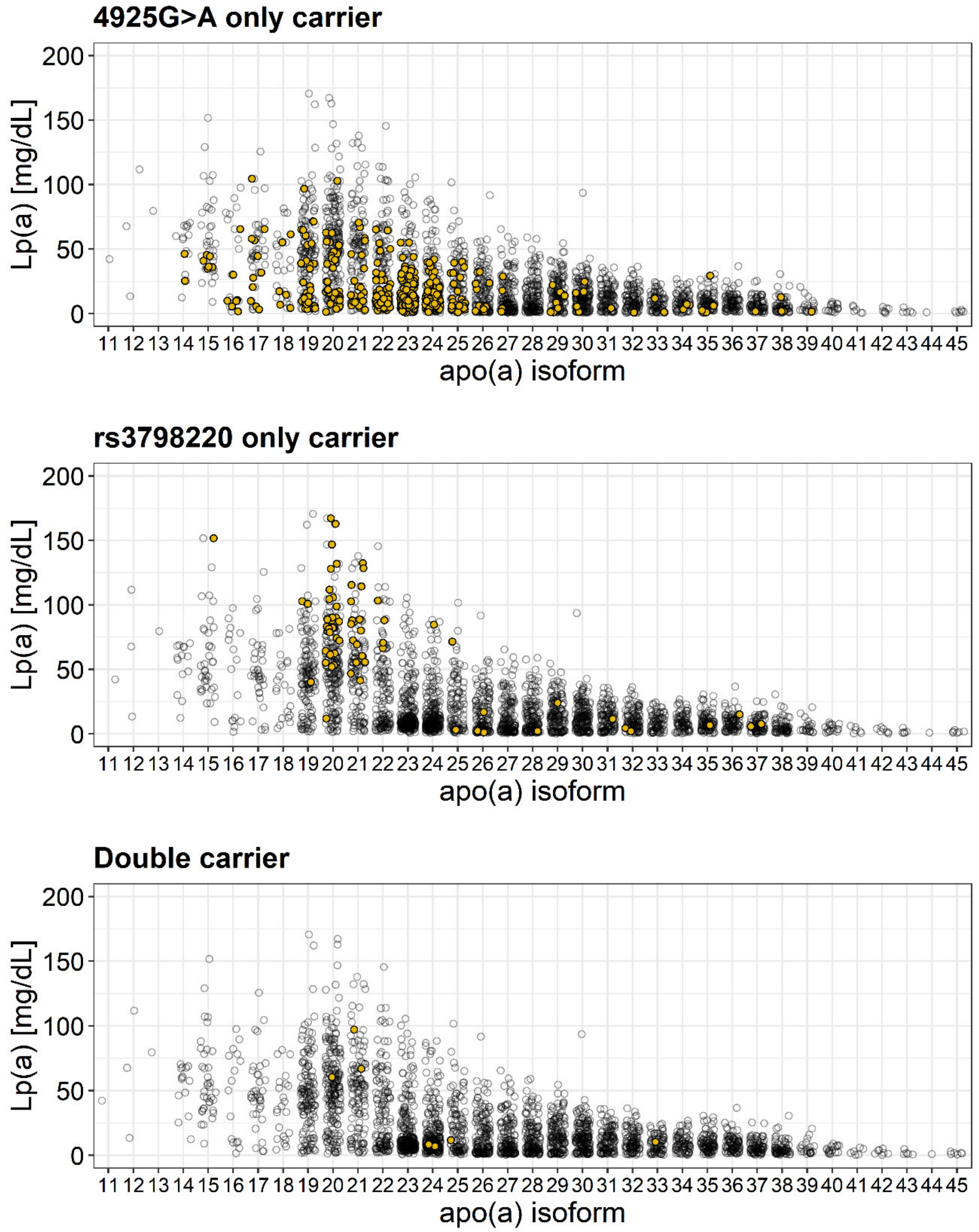

Supplementary Figure III. Lp(a) concentrations of different combinations of KIV-2 4925G>A and rs3798220 distributed over the different isoform sizes. Carriers per plot are highlighted in blue.

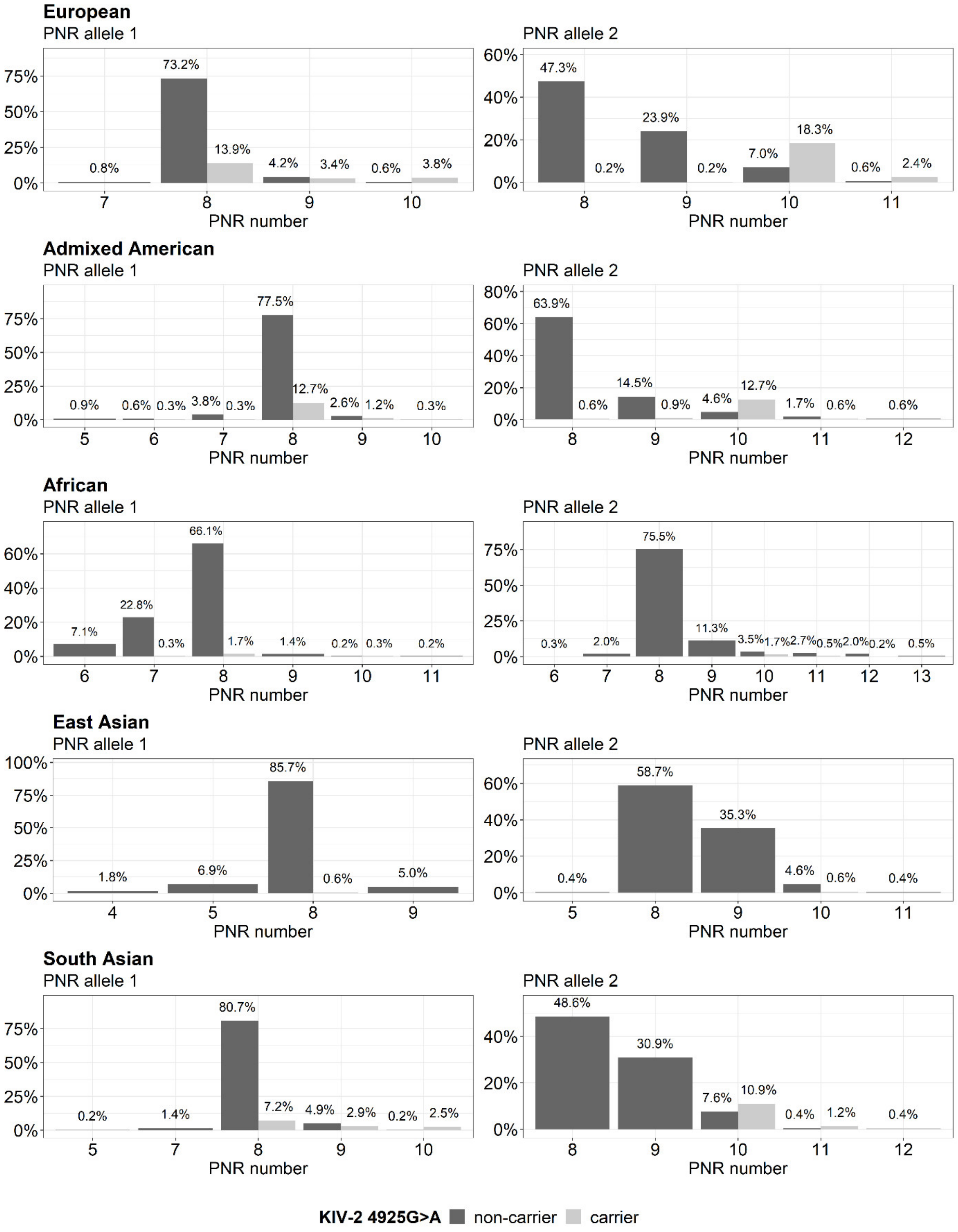

Supplementary Figure IV. Distribution of the short and the long PNR alleles (PNR alleles 1 and 2) in the superpopulations of the 1000 Genomes Project.

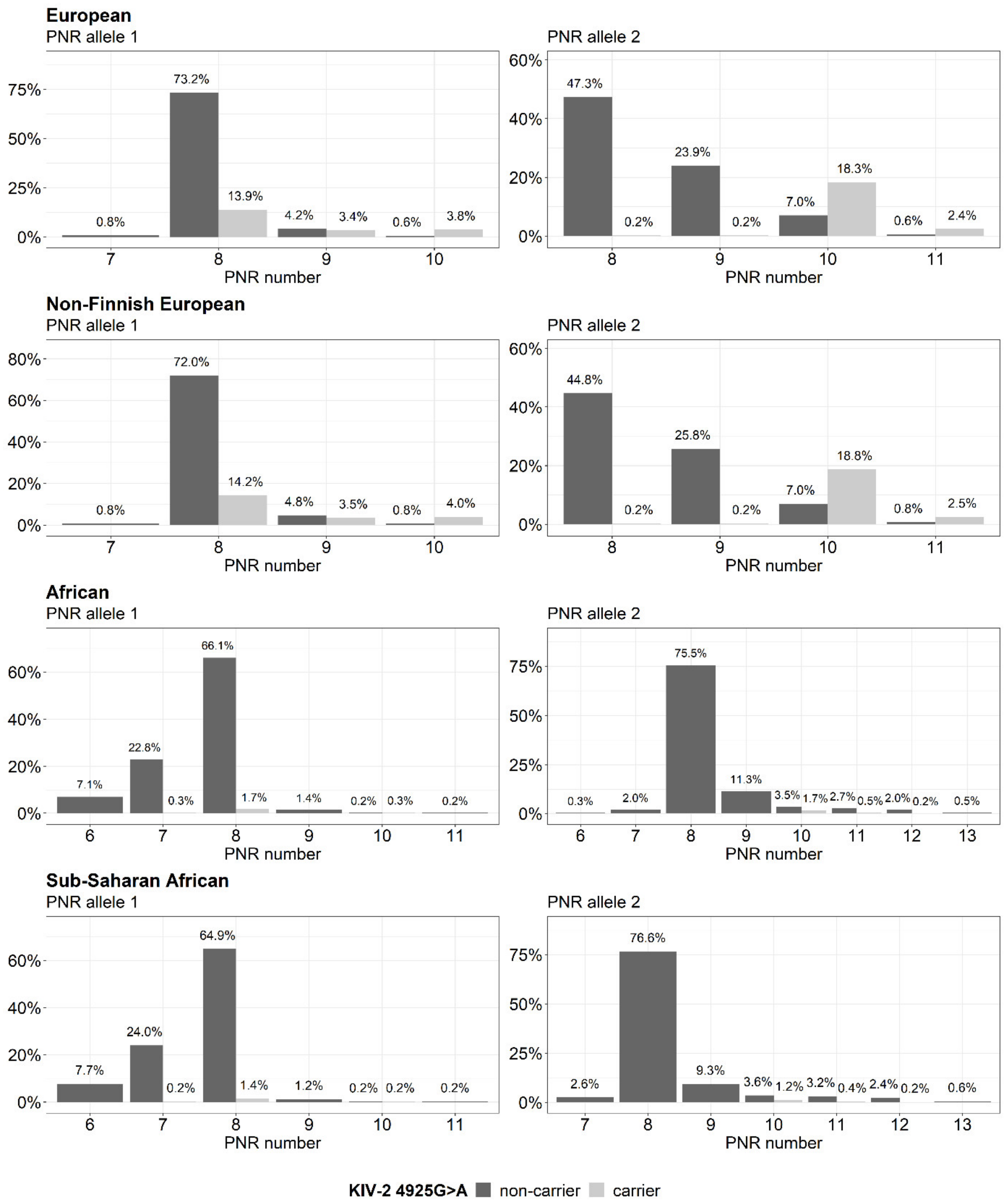

Supplementary Figure V. Distribution of the short and the long PNR alleles (PNR alleles 1 and 2) in the European, Non-Finnish European, African and Sub-Saharan African population. The Finnish population genetically differs from other European subpopulations (10,11), thus Non-Finnish Europeans individuals were extracted from the data for further analysis. The African 1000G superpopulation encompasses also groups with higher admixture rates from USA and Barbados (ASW, ACB). Thus only Sub-Saharan populations were regrouped here. Please note, that the bar plots for the African individuals and European individuals are the same as in Supplementary Figure IV. They are reproduced here to ease comparison of the Non-Finnish European and Sub-Saharan African subgroups with the respective 1000G superpopulation.

Supplementary Tables

Supplementary Table I. Quantile regression analysis of PNR allele 2 on Lp(a) concentration adjusted for PNR allele 1 (joint model). In all models PNR8 allele was used as reference allele. Effect given in mg/dL.

				**Model 1: adjusted for age and sex**			**Model 2: adjusted for age, sex and the smaller apo(a) isoform**		
	**variant**	**carrier [n]**	**Median Lp(a) concentration (IQR)**	**β**	**95% CI**	**p-value**	**β**	**95% CI**	**p-value**
**All**	PNR8	1,388	13.4 (4.8, 39.0)	reference					
	PNR9	648	11.1 (4.6, 23.7)	-2.02	-3.92, -0.13	0.0367	-2.76	-3.83, -1.68	5.17E-07
	PNR10	736	10.4 (6.2, 23.1)	-2.92	-4.48, -1.36	0.0003	-13.77	-15.35, -12.18	5.16E-62
	PNR11	84	10.9 (5.5, 19.6)	-1.61	-3.94, 0.72	0.1752	-15.95	-18.46, -13.45	7.70E-35
	PNR12	2	NA	NA	NA	NA	NA	NA	NA
**LMW**	PNR8	356	53.2 (40.3, 74.4)	reference					
	PNR9	99	49.2 (31.4, 64.6)	-5.79	-12.09, 0.51	0.0719	-4.67	-11.32, 1.98	0.0719
	PNR10	196	28.2 (10.8, 54.5)	-22.39	-27.54, -17.23	1.03E-16	-22.78	-28.28, -17.28	1.03E-16
	PNR11	48	12.5 (5.7, 23.9)	-30.97	-39.85, -22.08	1.85E-11	-34.43	-43.81, -25.04	1.85E-11
	PNR12	2	NA	NA	NA	NA	NA	NA	NA
**HMW**	PNR8	1,032	8.7 (3.6, 18.3)	reference					
	PNR9	549	9.4 (3.8, 17.3)	0.44	-0.97, 1.87	0.5382	0.53	-0.72, 1.78	0.4099
	PNR10	540	8.6 (5.3, 14.5)	-0.25	-1.35, 0.86	0.6614	-2.96	-3.97, -1.96	9.17E-09
	PNR11	36	9.2 (4.2, 17.3)	0.91	-2.77, 4.59	0.6273	-1.18	-4.08, 1.73	0.4266
	PNR12	0	NA	NA	NA	NA	NA	NA	NA

Supplementary Table II. Quantile regression analysis of PNR allele 2 on Lp(a) concentration adjusted for rs41272110 (joint model). In all models PNR8 allele was used as reference allele. Effect given in mg/dL.

				Model 1: adjusted for age and sex			Model 2: adjusted for age, sex and the smaller apo(a) isoform		
	**variant**	**carrier [n]**	**Median Lp(a) concentration (IQR)**	**β**	**95% CI**	**p-value**	**β**	**95% CI**	**p-value**
**All**	PNR8	1,388	13.4 (4.8, 39.0)	reference					
	PNR9	648	11.1 (4.6, 23.7)	-2.24	-4.08, -0.39	0.0174	-2.36	-3.63, -1.09	0.0003
	PNR10	736	10.4 (6.2, 23.1)	-5.10	-7.00, -3.20	1.46E-07	-8.74	-10.83, -6.65	3.89E-16
	PNR11	84	10.9 (5.5, 19.6)	-4.50	-7.21, -1.80	0.0011	-11.52	-14.64, -8.40	5.71E-13
	PNR12	2	NA	NA	NA	NA	NA	NA	NA
	rs41272110	744	11.1 (6.8, 25.0)	2.05	0.87, 3.23	0.0007	-6.62	-8.27, -4.98	3.84E-15
**LMW**	PNR8	356	53.2 (40.3, 74.4)	reference					
	PNR9	99	49.2 (31.4, 64.6)	-5.90	-12.35, 0.55	0.0733	-5.07	-11.89, 1.74	0.1448
	PNR10	196	28.2 (10.8, 54.5)	-17.73	-26.24, -9.22	4.97E-05	-16.86	-25.86, -7.86	0.0003
	PNR11	48	12.5 (5.7, 23.9)	-31.21	-42.84, -19.58	1.93E-07	-30.47	-42.79, -18.16	1.52E-06
	PNR12	2	NA	NA	NA	NA	NA	NA	NA
	rs41272110	239	21.1 (9.3, 51.4)	-8.65	-15.66, -1.63	0.0159	-9.00	-16.45, -1.55	0.0182
**HMW**	PNR8	1,032	8.7 (3.6, 18.3)	reference					
	PNR9	549	9.4 (3.8, 17.3)	0.45	-0.94, 1.83	0.5272	0.55	-0.75, 1.86	0.4074
	PNR10	540	8.6 (5.3, 14.5)	-2.21	-3.67, -0.76	0.0029	-3.14	-4.36, -1.91	5.78E-07
	PNR11	36	9.2 (4.2, 17.3)	-0.74	-2.74, 1.26	0.4657	-0.99	-4.02, 2.05	0.5243
	PNR12	0	NA	NA	NA	NA	NA	NA	NA
	rs41272110	505	9.4 (6.2, 16.5)	2.32	1.33, 3.30	4.35E-06	0.44	-0.52, 1.41	0.3669

Supplementary Table III. Median Lp(a) concentrations of carriers of rs10455872, rs3798220 and KIV-2 4925G>A. Carriers of rs10455872 or rs3798220 are defined as carriers of at least one minor allele copy. Lp(a) concentrations of single individuals per group are shown in Supplementary Figure II and III.

	**n (%)**	**median Lp(a), mg/dL (IQR)**
**Analyses on rs10455872 & 4925G>A**		
Wild type for both	1931 (67.6)	10.41 (3.88, 23.45)
rs10455872 only carrier	293 (10.3)	51.14 (39.82, 67.90)
4925G>A only carrier	592 (20.7)	9.21 (6.20, 14.93)
Carrier of both	42 (1.5)	46.99 (36.12, 60.03)
**Analyses on rs3798220 & 4925G>A**		
Wild type for both	2151 (75.3)	12.36 (4.48, 32.16)
rs3798220 only carrier	73 (2.6)	79.73 (53.23, 90.36)
4925G>A only carrier	627 (21.9)	9.62 (6.31, 17.39)
Carrier of both	7 (0.2)	11.69 (9.20, 63.82)

Supplementary Table IV. Carrier frequencies of KIV-2 4925G>A, PNR10 allele and PNR11 allele in the different populations in the 1000 Genomes Project.

Population	Description	total [n]	4925G>A carrier [n]	4925G>A carrier frequency [%]	PNR10 carrier [n]*	PNR10 carrier frequency [%]*	PNR11 carrier [n]*	PNR11 carrier frequency [%]*
**EUR**	European	497	105	21.1	126	25.4	15	3.0
CEU	Utah residents (CEPH) with Northern and Western European ancestry	98	23	23.5	29	29.6	1	1.0
FIN	Finnish in Finland	97	18	18.6	23	23.7	2	2.1
GBR	British in England and Scotland	90	25	27.8	25	27.8	5	5.6
TSI	Toscani in Italy	105	22	21.0	30	28.6	5	4.8
IBS	Iberian Population in Spain	107	17	15.9	19	17.8	2	1.9
NFE	Non-finnish European	400	87	21.8	103	25.8	13	3.3
**AMR**	Admixed American	346	51	14.7	60	17.3	8	2.3
MXL	Mexican Ancestry from Los Angeles USA	63	15	23.8	15	23.8	1	1.6
CLM	Colombians from Medellin, Colombia	94	16	17.0	20	21.3	2	2.1
PEL	Peruvians from Lima, Peru	85	10	11.8	10	11.8	1	1.2
PUR	Puerto Ricans from Puerto Rico	104	10	9.6	15	14.4	4	3.8
**AFR**	African	661	15	2.3	34	5.1	21	3.2
ASW	Americans of African Ancestry in SW USA	61	3	4.9	2	3.3	2	3.3
ACB	African Caribbeans in Barbados	96	3	3.1	8	8.3	1	1.0
GWD	Gambian in Western Divisions in the Gambia	113	5	4.4	6	5.3	7	6.2
YRI	Yoruba in Ibadan, Nigeria	108	2	1.9	6	5.6	4	3.7
MSL	Mende in Sierra Leone	85	1	1.2	4	4.7	0	0.0
ESN	Esan in Nigeria	99	1	1.0	4	4.0	1	1.0
LWK	Luhya in Webuye, Kenya	99	0	0.0	4	4.0	6	6.1
ssAFR†	Sub-Saharan African	504	9	1.8	24	4.8	18	3.6

Population	Description	total [n]	4925G>A carrier [n]	4925G>A carrier frequency [%]	PNR10 carrier [n]*	PNR10 carrier frequency [%]*	PNR11 carrier [n]*	PNR11 carrier frequency [%]*
**EAS**	East Asian	504	3	0.6	26	5.2	2	0.4
JPT	Japanese in Tokyo, Japan	104	3	2.9	7	6.7	2	1.9
CDX	Chinese Dai in Xishuangbanna, China	93	0	0.0	6	6.5	0	0.0
CHB	Han Chinese in Bejing, China	103	0	0.0	3	2.9	0	0.0
CHS	Southern Han Chinese	105	0	0.0	3	2.9	0	0.0
KHV	Kinh in Ho Chi Minh City, Vietnam	99	0	0.0	7	7.1	0	0.0
**SAS**	South Asian	486	61	12.6	90	18.5	8	1.6
STU	Sri Lankan Tamil from the UK	102	19	18.6	21	20.6	3	2.9
ITU	Indian Telugu from the UK	101	16	15.8	32	31.7	3	3.0
PJL	Punjabi from Lahore, Pakistan	94	10	10.6	11	11.7	0	0.0
GIH	Gujarati Indian from Houston, Texas	103	10	9.7	15	14.6	1	1.0
BEB	Bengali from Bangladesh	86	6	7.0	11	12.8	1	1.2

* PNR statistics derive from the longer PNR allele

† The ssAFR group excludes Americans of African Ancestry in Southwestern USA (ASW) and African Caribbeans in Barbados (ACB)

Supplementary Table V. Linkage disequilibrium statistics in different ethnicities from 1000 Genomes Project. Abbreviations for populations are shown in Supplementary Table IV.

					**4925G>A & PNR10**		**4925G>A & PNR11**	
**Population**	**total [n]**	**4925G>A carrier [n]**	**PNR10 carrier [n]**	**PNR11 carrier [n]**	**D'**	**R2**	**D'**	**R2**
**EUR**	497	105	126	15	0.84	0.76	0.75	0.27
**NFE**	400	87	103	13	0.83	0.76	0.71	0.26
**AMR**	346	51	60	8	0.85	0.77	NA	NA
**AFR**	661	15	34	21	0.72	0.47	0.18	0.15
**ssAFR**	504	9	24	18	0.65	0.40	0.19	0.14
**EAS**	504	3	26	2	NA	NA	NA	NA
**SAS**	486	61	90	8	0.85	0.69	0.71	0.25

References

1. Auton, A., Abecasis, G.R., Altshuler, D.M., Durbin, R.M., Abecasis, G.R., Bentley, D.R., et al. (2015). A global reference for human genetic variation. *Nature*. **526**, 68–74.

2. Li, H., Handsaker, B., Wysoker, A., Fennell, T., Ruan, J., Homer, N., et al. (2009). The Sequence Alignment/Map format and SAMtools. *Bioinformatics*. **25**, 2078–2079.

3. Byrska-Bishop, M., Evani, U.S., Zhao, X., Basile, A.O., Abel, H.J., Regier, A.A., et al. (2022). High-coverage whole-genome sequencing of the expanded 1000 Genomes Project cohort including 602 trios. *Cell*. **185**, 3426-3440.e19.

4. Mousavi, N., Shleizer-Burko, S., Yanicky, R., and Gymrek, M. (2019). Profiling the genome-wide landscape of tandem repeat expansions. *Nucleic Acids Res*. **47**, e90–e90.

5. Willems, T., Zielinski, D., Yuan, J., Gordon, A., Gymrek, M., and Erlich, Y. (2017). Genome-wide profiling of heritable and de novo STR variations. *Nat Methods*. **14**, 590–592.

6. Knaus, B.J., and Grünwald, N.J. (2017). vcfr: a package to manipulate and visualize variant call format data in R. *Mol Ecol Resour*. **17**, 44–53.

7. Okonechnikov, K., Golosova, O., Fursov, M., and team, the U. (2012). Unipro UGENE: a unified bioinformatics toolkit. *Bioinformatics*. **28**, 1166–1167.

8. Coassin, S., Erhart, G., Weissensteiner, H., Eca Guimarães de Araújo, M., Lamina, C., Schönherr, S., et al. (2017). A novel but frequent variant in LPA KIV-2 is associated with a pronounced Lp(a) and cardiovascular risk reduction. *Eur Heart J*. **38**, 1823–1831.

9. Weissensteiner, H., Forer, L., Fendt, L., Kheirkhah, A., Salas, A., Kronenberg, F., et al. (2021). Contamination detection in sequencing studies using the mitochondrial phylogeny. *Genome Res*. **31**, 309–316.

10. Salmela, E., Lappalainen, T., Fransson, I., Andersen, P.M., Dahlman-Wright, K., Fiebig, A., et al. (2008). Genome-Wide Analysis of Single Nucleotide Polymorphisms Uncovers Population Structure in Northern Europe. *PLoS One*. **3**, e3519.

11. Lao, O., Lu, T.T., Nothnagel, M., Junge, O., Freitag-Wolf, S., Caliebe, A., et al. (2008). Correlation between Genetic and Geographic Structure in Europe. *Curr Biol*. **18**, 1241–1248.

12. Tucci, S., and Akey, J.M. (2019). The long walk to African genomics. *Genome Biol*. **20**, 130.
